# Stereochemical Study of the Super Large Tetrakis Alkaloid Alasmontamine A by Means of an Advanced Computational NMR

**DOI:** 10.3390/ijms24065572

**Published:** 2023-03-14

**Authors:** Valentin A. Semenov, Leonid B. Krivdin

**Affiliations:** A. E. Favorsky Irkutsk Institute of Chemistry, Siberian Branch of the Russian Academy of Sciences, Favorsky St. 1, 664033 Irkutsk, Russia

**Keywords:** computational ^1^H and ^13^C NMR, DFT, alasmontamine A, DP4+, natural products

## Abstract

^1^H and ^13^C NMR chemical shifts of the tetrakis monoterpene indole alkaloid alasmontamine A, with a molecular formula of C_84_H_91_N_8_O_12_, have been calculated within the DFT framework. Six minimum energy conformers of this alkaloid were identified, and three key configurations that contribute to its NMR shielding constants were established. Several ambiguities in the reported assignment of the NMR chemical shifts of alasmontamine A have been resolved.

## 1. Introduction

**Figure 1:**
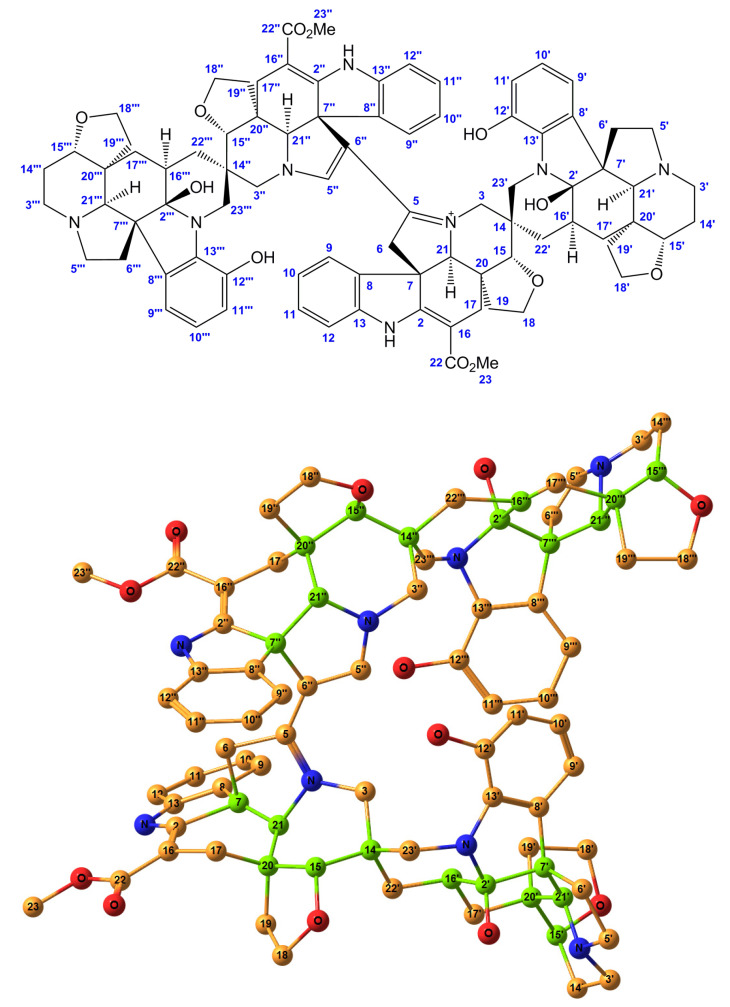
**Numbering scheme (top) and the stereochemical structure (bottom)** of alasmontamine A; for clarity, hydrogen atoms are not shown. Element colors are as follows: carbons—yellow, nitrogens—blue, and oxygens—red, while all 22 asymmetric carbons are shown in green.

Theoretical calculation of ^13^C NMR chemical shifts provides a powerful tool in the structural elucidation of organic molecules, natural products, carbohydrates, and biochemical species [1,2,3]. Continuing our very recent survey of large natural products with multiple asymmetric centers, [4,5,6,7,8,9] the current study is focused on a tetrakis monoterpene indole alkaloid, alasmontamine A, Figure 1. The 2D chemical structure of this large alkaloid providing the enumeration of atoms (with omitting hydrogens for clarity) is provided below, while its 3D structure is presented in Figure 1. A preliminary communication dealing with the conformational study of this compound was published in *Magnetic Resonance in Chemistry* [10].

Historically, in the search for structurally and biogenetically interesting alkaloids from tropical plants found in Indonesia, the first tetrakis monoterpene indole alkaloid, alasmontamine A, consisting of the tetrakis moieties, was isolated and identified by Hirasawa and coworkers [11]. The molecule of alasmontamine A consists of 18 *sp*^2^ and 12 *sp*^3^ quaternary carbons, 15 *sp*^2^ and 10 *sp*^3^ methines, 27 *sp*^3^ methylenes, and 2 methyl groups, providing in total 22 asymmetric centers (shown in green in Figure 1), resulting in a molecular formula of C_84_H_91_N_8_O_12_. The structure of this molecule was thoroughly elucidated by means of COSY, HOHAHA, HSQC, and HMBC experiments using a 920 MHz NMR spectrometer [11].

For alasmontamine A, containing four monoterpene indole subunits, the inner rotation of two conjugated parts around the C5−C6″ bond results in an enormous difficulty in establishing the correct rotational conformation around this bond. Nevertheless, despite these obvious difficulties, Hirasawa et al. have managed to establish experimentally the relative stereochemistry together with the conformational features of alasmontamine A using advanced NMR techniques. In particular, they found that two main parts (designated by the authors as *A* and *B*) adopted a predominantly *synclinal* orientation to each other, while four piperidine moieties at the two *spiro* carbons, C-14 and C-14″, had the *boat–chair* and *chair–chair* conformations, respectively.

Undoubtedly, the quantum chemical reconstruction of the experimental three-dimensional structure of this challenging natural product, including more than 100 atoms of the 2nd row elements, is a very non-trivial task. We have attempted to recreate the original liquid phase geometry of alasmontamine A and to perform a calculation of the ^1^H and ^13^C NMR chemical shifts of the deduced true minimum energy conformers using the most effective DFT computational protocol, derived in our recent studies of strychnine and derivatives.

For example, it is well known that the calculation of NMR shielding constants in combination with the probabilistic DP4 approach by Smith and Goodman [12] is a powerful tool for determining the stereochemistry of natural products when only one set of experimental data is available. In addition, the modification of the DP4 method, known as DP4+ [13], is much more efficient in making correct NMR signal assignments with a better confidence than the probabilities based only on the scaled chemical shifts. In the case where the structure of a natural product or synthetic compound providing several asymmetric centers results in a very similar spectral pattern, the general formalism of the DP4 method and its most recent developments, such as DP4+ and DP4-*J* [14], as well as a combination of theoretically calculated NMR parameters and artificial neural networks [15,16,17], are very robust and more reliable, as compared to classical statistical methods. These approaches to the calculation of the NMR parameters of natural products and complex synthetic compounds with multiple chiral centers are described in detail in the most recent reviews by Marcarino et al. [18] and Costa et al. [19].

DP4+ calculations performed at a higher level of theory lead to more reliable results compared to those obtained with DP4. It is interesting to note that when constructing correlations of linearly scaled calculated and experimental chemical shifts, an “insidious” phenomenon is sometimes observed, namely, when data corresponding to an irregular structure can randomly correlate better with the experiment. This is due to the fact that when leveling a systematic error according to the linear regression equation, the incorrect structures can have an unpredictably better correlation with the experimental data than the correct ones. This leads to the fact that the magnitude of error in calculating the scaled chemical shifts no longer depends on a number of factors that determine the total systematic error, including the stereoelectronic structure of the investigated molecule. This problem has long been noted by us in practical calculations of NMR chemical shifts and has been repeatedly noted by many others [19,20,21].

In this respect, we have focused our attention on the solution of this problem, which was originally formulated by Sarotti and coworkers [13], who added chemical shifts in its “pure” unscaled form to the original Goodman’s DP4 equation [12]. The influence of the stereoelectronic structure, including hybridization, was introduced as affecting the final result. At this, the term *µ*DP4+ was decomposed according to hybridization due to the fact that the multiplicity of unscaled errors did not correspond to Student’s *t*-distribution, and accordingly, its median was not centered at zero [13]. It should also be recalled that in the development of the DP4+ method, in addition to *ν* and *σ* (both scaled), as many as six parameters were introduced for each type of nucleus, namely, *ν* (unscaled-*sp*^2^), *μ* (unscaled-*sp*^2^), *σ* (unscaled-*sp*^2^), *ν* (unscaled-*sp*^3^), *μ* (unscaled-*sp*^3^), and *σ* (unscaled-*sp*^3^); see Section 3.4 for details.

In this work, we applied a general DP4 scheme in its DP4+ modification to a molecule of the tetrameric bisindole alkaloid alasmontamine A containing as many as 22 asymmetric centers, which are marked in green in Figure 1.

## 2. Results and Discussion

In the present paper, we used a general workflow methodology, which was previously proposed by us in the study of stereochemically rich diverse natural products [22].

**Scheme 1:**
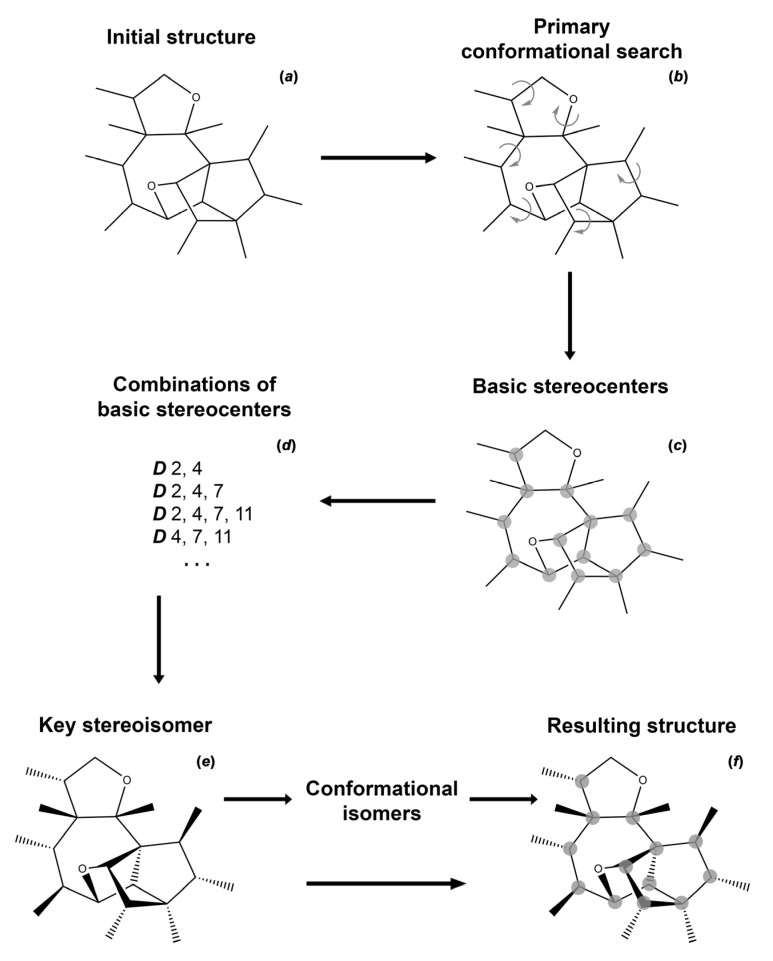
A general workflow for the establishing of stereochemical structure of natural products with multiple asymmetric centers. Steps: (**a**)—originally established structure (all experimental data are taken into account); (**b**)—structure established during the primary conformational search; (**c**)—identification of asymmetric centers; (**d**)—establishment of all basic diastereomers; (**e**)—identification of the key stereoisomer; (**f**)—establishment of the final configuration with taking into account conformational features.

To date, several protocols have been suggested to establish the stereochemistry of natural products based on the accurate high-level calculation of their NMR chemical shifts [13,23,24]. In the present study, we used a simple and reliable scheme to unambiguously establish the stereochemical structure of natural products, especially of those in difficult and/or ambiguous cases. This scheme, the algorithm of which is presented in Scheme 1, is based on the use of a set of powerful statistical tools for the analysis of calculated isotropic shielding constants and the corresponding chemical shifts of a number of diastereomers and rotational conformers.

The proposed workflow involves several computational procedures, including the initial conformational search, the search of basic and key diastereomers, and the establishment of their conformations. First of all, the initial structure established using a set of NMR techniques was taken as the starting point of the study. For this structure, a primary low-level conformational search was carried out, resulting in the groups of the most energetically preferable conformers being determined. In this case, all groups of conformers must satisfy the experimentally obtained data on the stereochemical structure of the molecule, including the results of NOESY, ROESY, and other pulse sequences. From the entire set of potential conformers, one candidate was selected from the group of energetically more stable ones, which better satisfies the results of the NMR experiment. Further, the geometry of this conformer was subjected to further reoptimization at a higher level of theory. After that, all the asymmetric centers of the molecule were established and the basic diastereomers were determined, namely, the stereoisomers with a changed configuration at each stereocenter.

An additional criterion for selecting a set of basic diastereomers can be an estimate of their Gibbs free energy. Obviously, the probability of the existence of stereoisomers with ∆*E* greater than 10–20 kcal/mol from the minimum is extremely small. However, in the natural environment, the predominance of less energetically favorable stereoisomers of natural products with multiple asymmetric centers is quite possible.

Based on the selection of basic stereocenters whose configuration change does not lead to a significant decrease in the correlation of theoretical and experimental chemical shifts, all combinations of diastereomers at these asymmetric centers are constructed. It is important to note that at this stage, there is no need to consider all possible 2*^n^* stereoisomers of the molecule, but it can be limited to only 2*^m^* diastereomers with *m* = 4–6. Such a significant reduction in the number of considered stereoisomers is associated with the fact that, as a rule, a change in the configuration at the same time for more than 4–6 asymmetric centers is most unlikely for the majority of organic molecules of up to 50–100 atoms of the second period.

Similarly to the previous step, among the 2*^m^* diastereomers, one (maximum two in the case of an ambiguous result) key diastereomer was selected based on the results of the performed correlation analysis, the configuration of the chiral centers of which, as expected, corresponds to the *genuine*. In addition, to enhance the reliability of the proposed method within the framework of the general scheme of the statistical analysis, the formalism of the DP4+ approach or similar can be applied, which allows for estimating the probability distribution of each of the 2*^m^* studied stereoisomers.

Based on the described methodology, in order to establish the three-dimensional structure of alasmontamine A, a preliminary conformational search of this huge alkaloid was conducted. In particular, the structural features of two principal *spiro* carbons, C-14 and C-14″, were thoroughly examined. At that, the available NOESY correlations between the furan and phenolic fragments of the second and fourth subunits were taken into account. The performed conformational search was focused mainly on finding the exact angle of rotation around the C-5–C-6″ bond. In the process of a conformational search in the range of 0–25 kcal/mol, several groups of conformers were identified, fully, or partially corresponding to the stereochemical composition of alasmontamine A, as was originally established by means of different NMR experiments [11].

**Figure 2:**
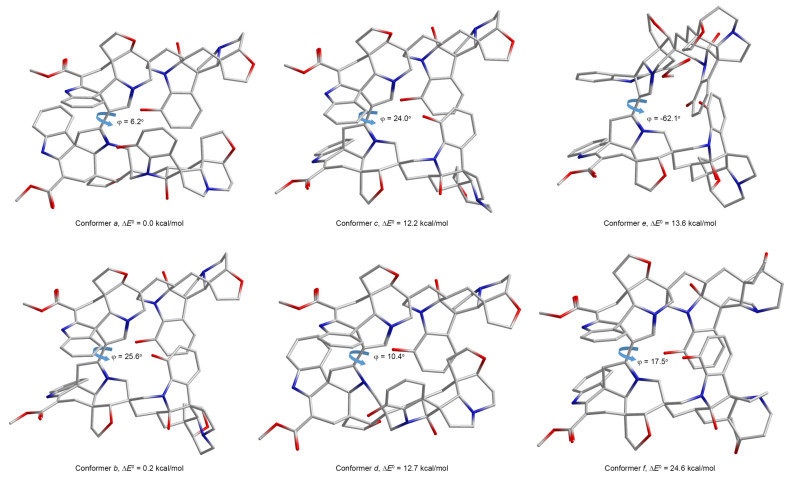
Three-dimensional structures of the conformers ***a*–*f*** of alasmontamine A, optimized at the M06-2X/aug-cc-pVTZ level. Indicated dihedral angles *φ* describe the internal rotation of two subunits around the C-5‒C-6″ bond. Adopted with minor editing privilege from the preliminary publication by Semenov and Krivdin [10].

As a result of this computational scrutiny, six conformers of alasmontamine A (***a***–***f***) were selected from each of the established groups, which are shown in Figure 2. For each of these six low-energy conformers, all ^1^H and ^13^C NMR chemical shifts were calculated on the geometries, optimized at the M06-2X/cc-pVTZ//aug-cc-pVTZ level of theory.

**Figure 3:**
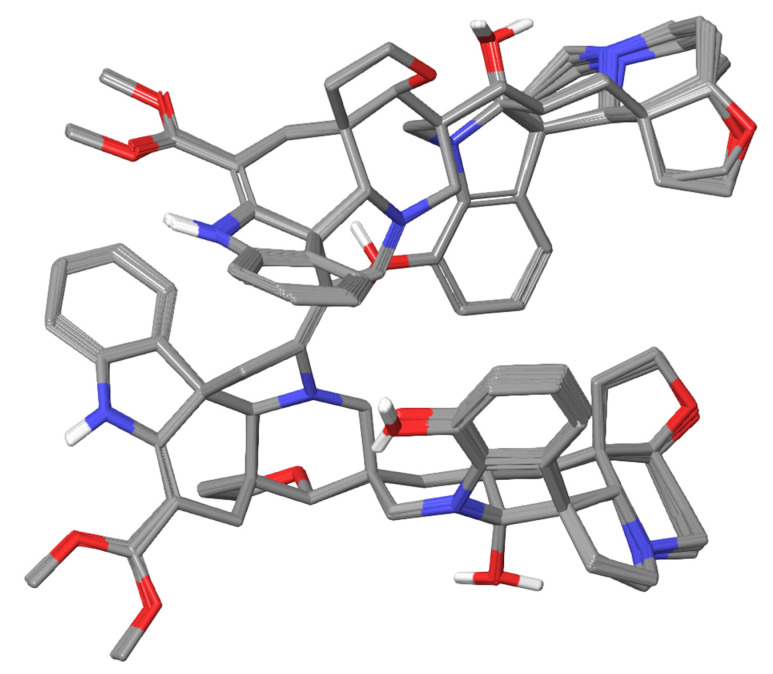
Superimposed structures of a set of the low-energy conformers of group ***b*** obtained by conformational search throw.

In the preliminary communication of this study [10], conformer ***b*** was determined to be the most preferable as one, providing the most accurate correlations of the calculated and experimental ^1^H and ^13^C NMR chemical shifts. Moreover, among the six selected conformers ***a***–***f***, conformer ***b*** was found to be one of the most energetically favorable ones. The superimposed structures of a set of low-energy conformers belonging to group ***b*** are shown in Figure 3.

**Figure 4:**
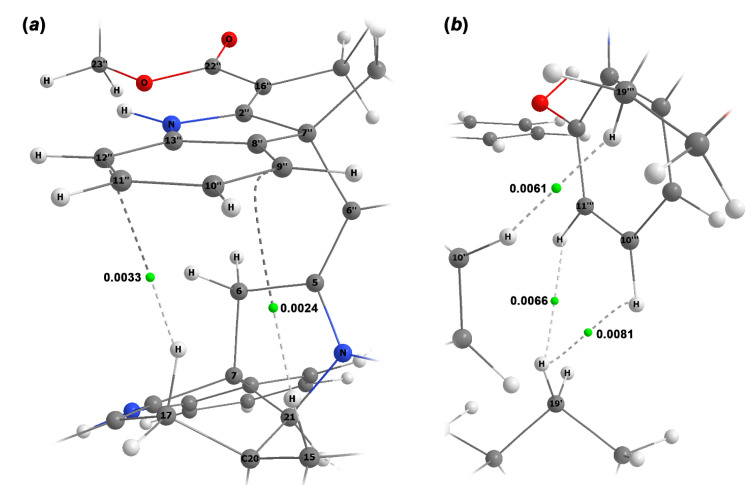
Spatial arrangement of principal non-valent intramolecular interactions of the first and third (**a**), and second and fourth (**b**) subunits of alasmontamine A together with (3,‒1) bond critical points (the latter are shown as green circles) with corresponding numerical values of electron density (a.u.).

As can be seen, the largest conformational lability occurs in the furoindolizine fragments of the second and fourth subunits of alasmontamine A. This fact characterizes their relative stereochemical mobility since they are sufficiently away from the rigid binding framework C6-C5-C6″-C5″ of this molecule. This finding was also confirmed by the results of the performed topological analysis presented in Figure 4. At this stage, to prove the presence of all intramolecular non-valent contacts, a topological analysis was carried out in the framework of the Bader QTAIM approach [25].

In this case, the wave function was obtained for conformer ***b***, the latter considered in its optimized geometry. As a result of the performed analysis of this wave function, all (3,–1) the bond critical points (BCP) were revealed, which fully corresponded to the experimental data of the two-dimensional NOESY correlations. In particular, the anisotropic effect of the C-8″–C-13″ aromatic system was confirmed.

The presence of a *t*-stacking was also manifested, the latter determined by two BCPs, namely, those between C-9″, C-12″, and H-15, H-17*β*; see Figure 4a. As a result of a well-pronounced *t*-stacking, the signals of these protons were shifted to higher field, as compared to their counterparts H-15″, H-17*β*″. On the other hand, a rather unusual manifestation of the non-valent interaction between protons of different subunits was also observed, which followed from the presence of BCP between the protons H-10′ and H-19″, as well as those between the *α-*proton at C-19′ and the protons H-10‴, H-11‴ of the aromatic moiety C-8″–C-13″ (Figure 4b). It appeared that the results of the performed topological analysis were in full agreement with the experimental NMR data.

**Figure 5:**
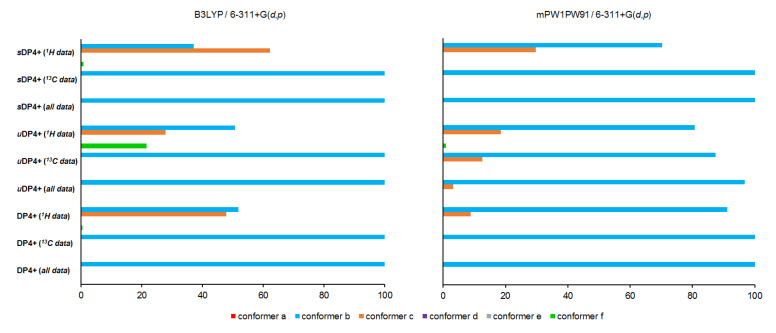
Graphs of the DP4+ probability distribution (%) of the conformers of alasmontamine A, obtained by correlating calculated vs. experimental NMR data. Minor isomers with a molar fraction of less than 1% are not visible on the diagram. *s*DP4+ denotes scaled values of DP4+; *u*DP4+ denotes unscaled values of DP4+ (see Section 3.4 for details).

For an enhanced reliability in choosing the correct conformer, we have performed the correlation analysis of six groups of conformers using the DP4+ approach. Within the framework of this methodology, we compared two computational schemes for estimating NMR shielding constants using the B3LYP and mPW91PW91 functionals in combination with the 6-311G(*d*) basis set; see Figure 5.

As one can see, the resulting probability of the predominant conformer ***b*** is almost 100% for both computational schemes. However, in both cases, there is some uncertainty in the choice between conformers ***b*** and ***c*** based solely on theoretical dataset of their ^1^H NMR scaled constants. The same uncertainty is present in the B3LYP/6-311G(*d*) computational scheme, which employs a set of the unscaled ^1^H NMR data. Taking into account this ambiguity, we decided to use the mPW91PW91/6-311G(*d*) computational scheme in all further calculations of the NMR chemical shifts of the stereoisomers of alasmontamine A.

Using conformer ***b*** as the basic starting point, all “main” diastereomers were created in that way. In line with the suggested here workflow methodology, the configuration of alasmontamine A was changed “step by step” across all 22 asymmetric centers. Thus, for 22 stereoisomers with a stepwise change relative to the initial configuration, the geometric parameters were optimized, and corresponding ^1^H and ^13^C NMR chemical shifts were calculated.

**Figure 6:**
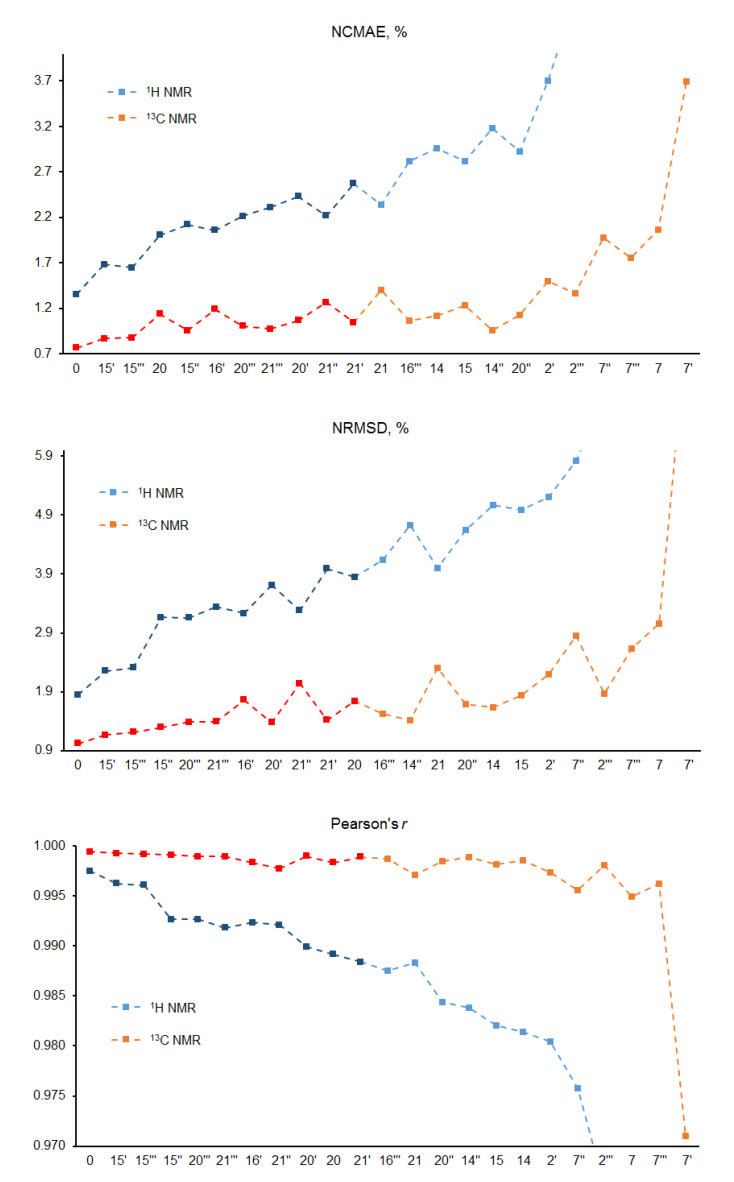
Normalized CMAE (%), normalized RMSD (%), and Pearson’s correlation coefficient for the “main” diastereomers of alasmontamine A.

Using an array of experimental spectral data together with the corresponding set of theoretical data, a correlation-based estimation of all the main diastereomers of alasmontamine A was carried out by taking into account such statistical descriptors as the corrected Mean Absolute Error (CMAE), Root-Mean-Square Deviation (RMSD), together with Pearson’s correlation coefficient, evaluated using Equations (3)–(5), respectively, as presented in Figure 6.

Based on the deviations of these descriptors, as many as six principle stereocenters of alasmontamine A were established. The alteration of the configuration of each of those optical centers was close to the range of the confidence interval in the whole series of diastereomers under consideration. Namely, the following principle stereocenters were considered: C-15′, C-15‴, C-15″, C-21‴, C-16′, and C-21′. The configuration of C-20‴ was not included in this set, which was because it did not match experimental NOESY data due to the lack of the H-10′ and H-19‴ correlation.

**Figure 7:**
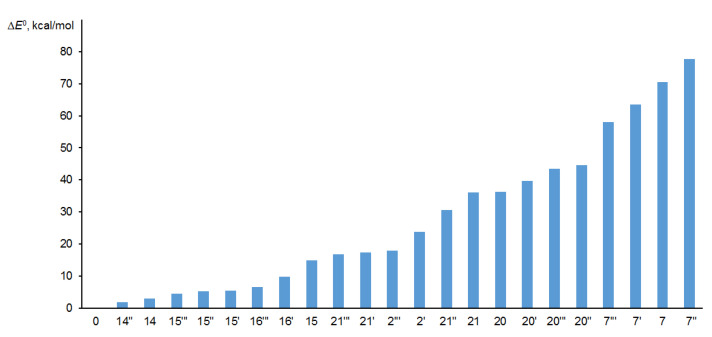
Difference of the absolute energies Δ*E*^0^ (kcal/mol) of the main diastereomers of alasmontamine A, as compared to the original configuration.

An additional criterion for selecting the set of key stereocenters can be the estimate of their Gibbs free energies. As it has been mentioned above, the probability of the existence of stereoisomers with Δ*E*^0^ > 20 kcal/mol from the global minimum is vanishingly small. Therefore, configurations such as C-21″, C-20′, and C-20 were also discarded, since their Gibbs free energies obtained by optimizing the corresponding molecules exceeded at least 30 kcal/mol, as compared to the structure with a basic configuration “0”, as shown in Figure 7.

In this line, as many as *N* = 2^6^ = 64 “alternate” diastereomers were constructed by varying configurations at six asymmetric centers, and the optimization of the geometric parameters together with the calculation of NMR chemical shifts was performed in each particular case. It appeared that the consideration of 6 out of 22 stereocenters was a necessary and sufficient condition for the reliable determination of the true configuration of the molecule of alasmontamine A.

**Figure 8:**
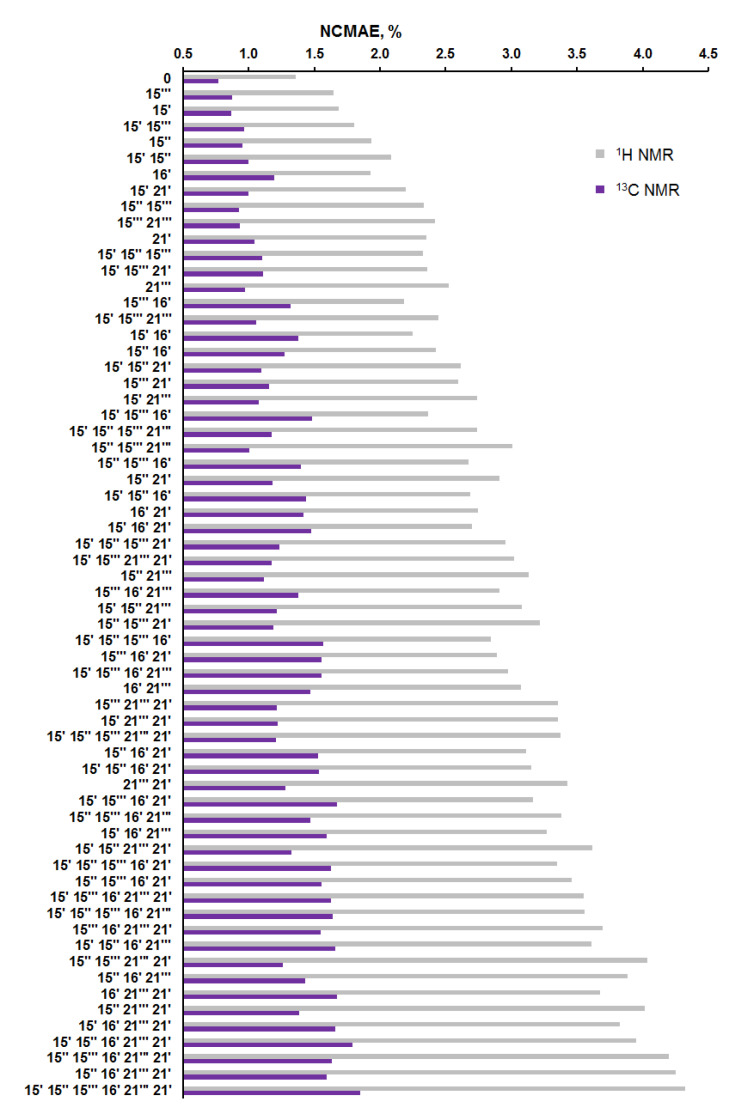
Distribution of the normalized CMAE values (%) of 64 alternate diastereomers of alasmontamine A.

**Figure 9:**
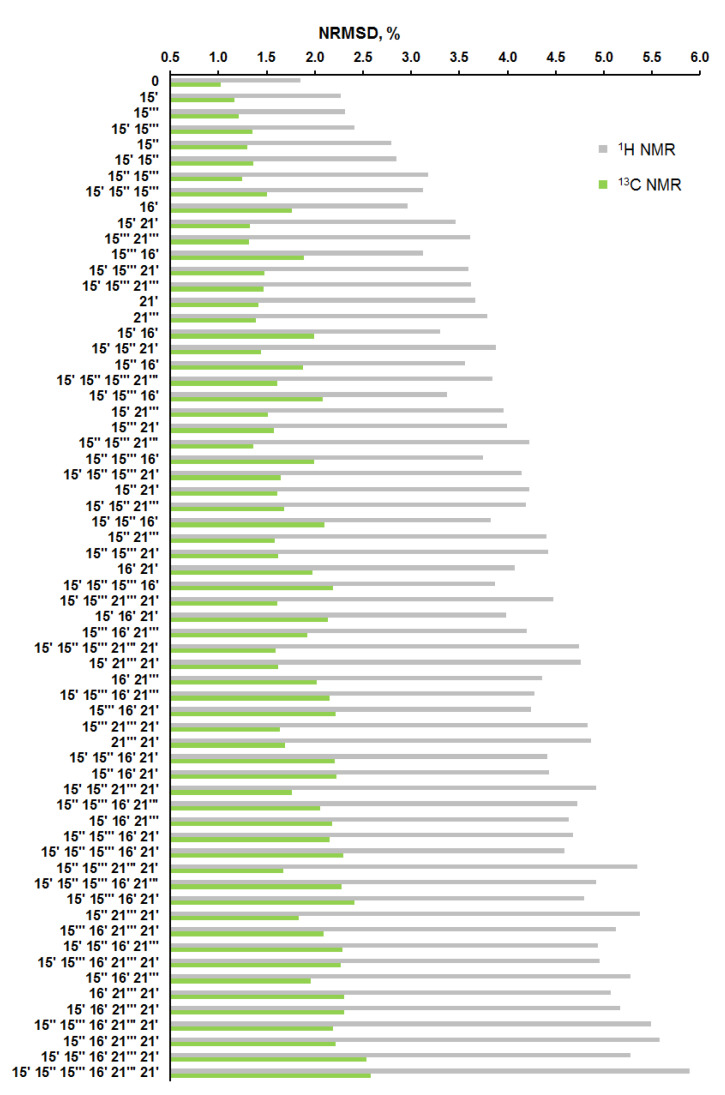
Distribution of the normalized RMSD values (%) of 64 alternate diastereomers of alasmontamine A.

**Figure 10:**
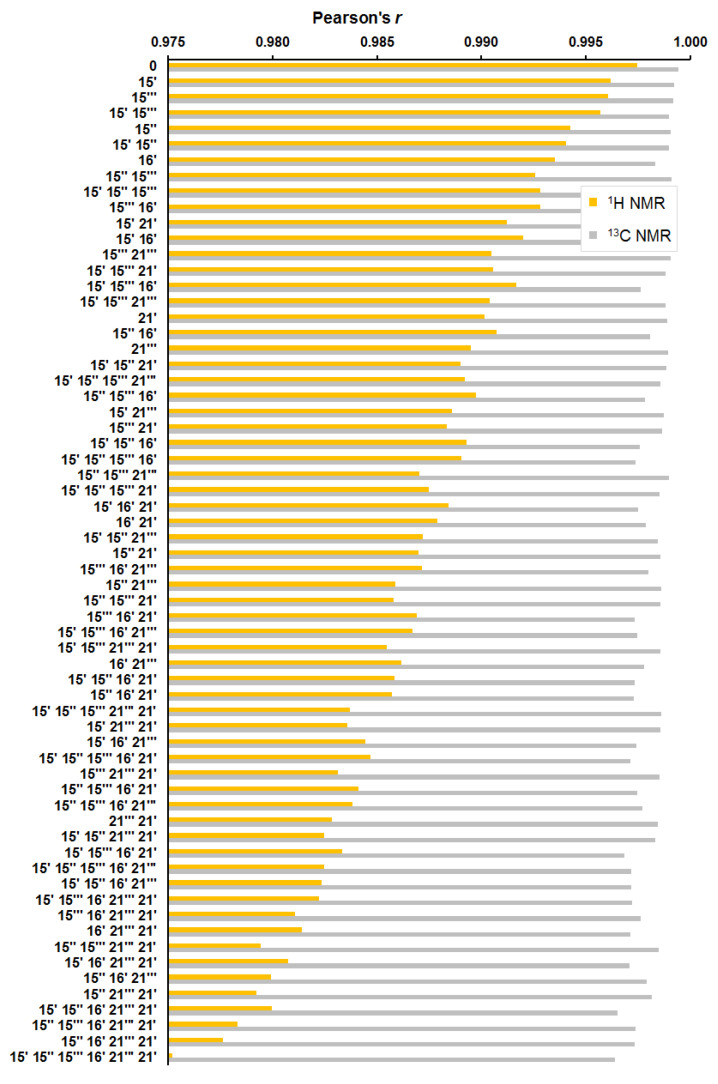
Distribution of Pearson’s correlation coefficient of 64 alternate diastereomers of alasmontamine A.

As in the previous step, for the considered 64 diastereomers, an estimation of the NMR parameters was carried out based on the calculated values of chemical shifts in the whole set of the conformers, providing a total of 10 560 NMR chemical shifts. It was found that diastereomers with an alternated configuration at the C-15‴ and C-15′ stereocenters had a close deviation from the original “0” configuration, as follows from the data shown in Figure 8, Figure 9 and Figure 10.

**Figure 11:**
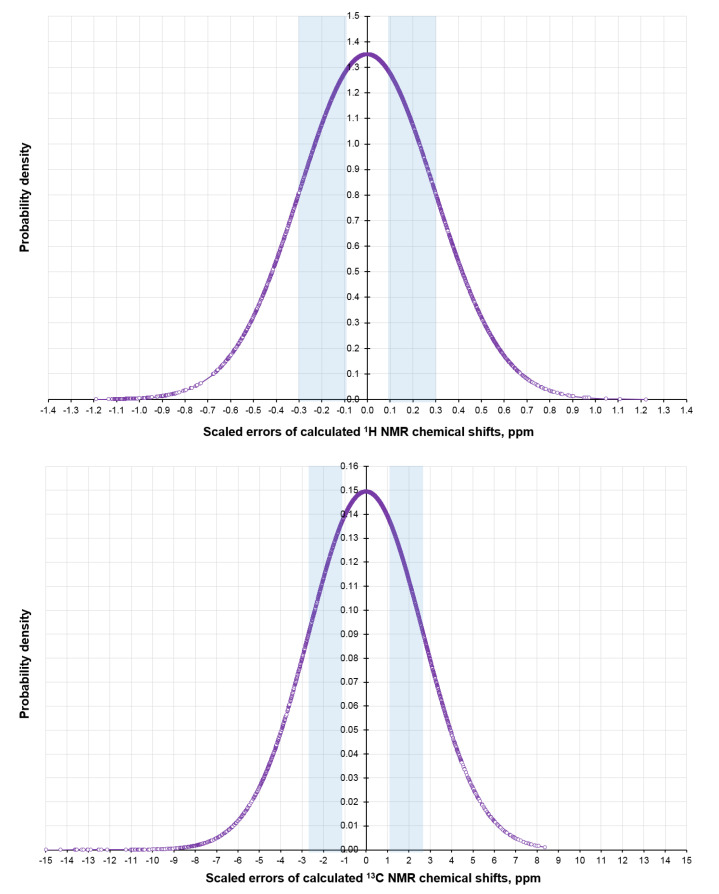
Normal distribution plots of scaled ^1^H (top) and ^13^C (bottom) NMR chemical shift errors.

In order to estimate the probability fractions of the studied set of diastereomers based on the scaled CMAE of ^1^H and ^13^C NMR chemical shifts, their normal distributions were also plotted, as shown in Figure 11.

However, the scatter range of the average CMAE values for 64 diastereomers turned out to be very narrow and amounted to only 0.09–0.30 ppm for the proton shielding constants and 1.1–2.7 ppm for the carbon ones. Therefore, the probability distribution for the alternate diastereomers calculated from the normal distribution of CMAE did not clarify the situation (see Figure S1 in the Supplementary Materials). The integration probabilities *P* [^1^H;^13^C] of the first five diastereomers appeared to be extremely close, being within the 2.0–2.2% range.

**Figure 12:**
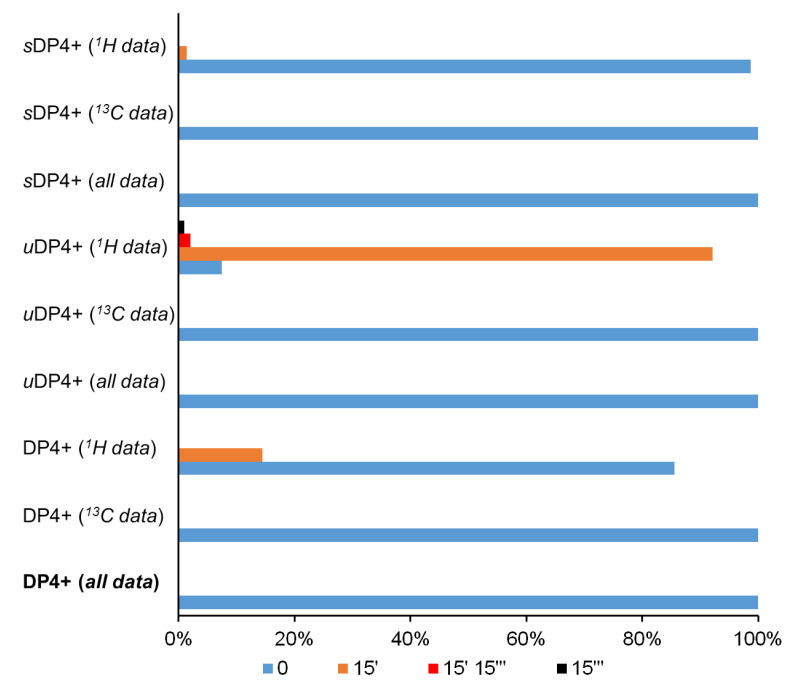
Graphs of the DP4+ probability distribution (%) of the diastereomers of alasmontamine A, obtained by correlating calculated vs. experimental NMR data. Minor isomers with molar fractions of less than 1% are not visible on the diagram.

This fact also manifests itself in the analysis of the key diastereomers within the DP4+ formalism. Indeed, the unscaled ^1^H constants clearly indicate on the predominance of the 15′ diastereomer, as follows from the data presented in Figure 12. However, the resulting probability of the basic configuration “0” was found to be dominant.

The main result of the performed study is that as many as three key configurations contribute to the resulting NMR shielding constants, namely, the original “0” configuration and two alternative configurations, modified at the C-15‴ and C-15′ stereocenters, with a clear predominance of the former. However, it should be noted that there are several ambiguous points in theoretical values of NMR chemical shifts, as presented in Table 1.

A complete set of data on all the calculated NMR spectral parameters of the diastereomers of alasmontamine A is provided in the Supplementary Materials. It follows that in the case of H-3*α* and H-9 protons, the non-valence interactions involving oxygen LEPs at C-12‴ and aromatic system C8″–C13″, correspondingly, were not taken into account in full. The protons H-18*α*, H-21, H-17″*β*, and H-23‴*α* are able to form solvate contacts, which is also not taken into account in full in the framework of the performed calculations. On the other hand, there are significant discrepancies with the experiment for the atoms of the aromatic systems C8′–C13′ and C8‴–C13‴. Apparently, this may be due to the stereoelectronic effects originating in the *π*-staking between these systems, which are not adequately reproduced at the DFT level.

**Figure 13:**
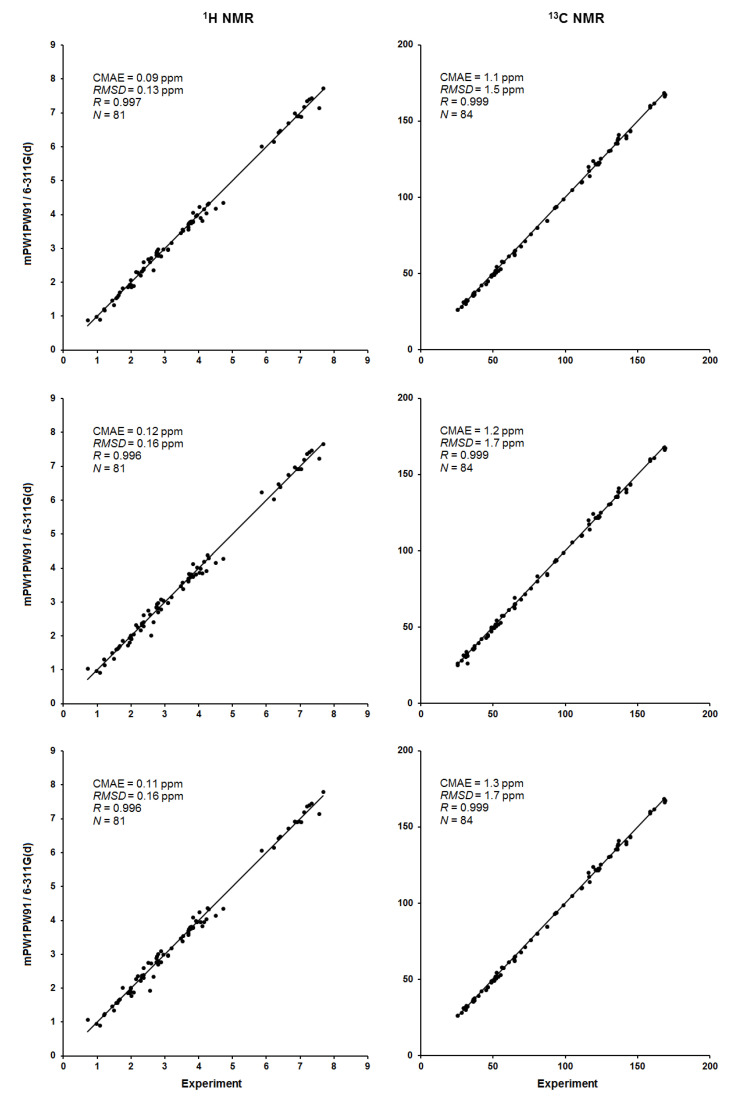
Correlation plots of calculated vs. experimental ^1^H and ^13^C NMR chemical shifts, ppm (**left** and **right**, respectively) for the key configurations 0 (**top**), 15′ (**middle**), and 15‴ (**bottom**) of alasmontamine A.

As a final illustration, presented below are the correlation plots between the theoretical and experimental values of ^1^H and ^13^C NMR chemical shifts for the three key configurations of alasmontamine A, as presented in Figure 13. It follows that calculated ^1^H and ^13^C NMR chemical shifts demonstrate a reasonably good agreement with the available experimental data, characterized by Pearson’s coefficient *R* being of about 0.996–0.999. It is seen that the most accurate correlations are observed for carbons. For all key configurations, the corresponding statistical parameters are almost identical. The final correlations are characterized by a CMAE of only 0.09–0.12 ppm for the range of about 7 ppm for protons and 1.1–1.3 ppm for the range of about 150 ppm for carbons.

## 3. Materials and Methods

### 3.1. Conformational Search and Geometry Optimization

The initial conformational search of alasmontamine A was performed using the OPLS3 force field in the liquid phase of methanol using the MacroModel program implemented in the Schrödinger Maestro 11.5 package [26]. In the course of this search, as many as 10^5^ steps were taken to find the most probable conformers. After that, the unique conformations of alasmontamine A were identified and subjected to a further geometry optimization, with GAUSSIAN 09 [27] at the M06-2X/cc-pVTZ//aug-cc-pVTZ level (diffuse functions were used only on nitrogen and oxygen atoms to take into account the effect of their multiple lone pairs). The solvent effect of methanol was accounted for within the Integral Equation Formalism Polarizable Continuum Model (IEF-PCM) [28,29]. All the considered optimized structures of alasmontamine A were found to be the true minima on the potential energy surface (PES), as was confirmed by the absence of imaginary frequencies at that computational level.

### 3.2. Calculation of Chemical Shifts

All calculations of ^1^H and ^13^C NMR isotropic magnetic shielding constants (the latter being converted into chemical shifts) were carried out at the DFT level in the liquid phase of methanol by implying the GAUSSIAN 09 code. In these calculations, we used the one-parameter hybrid functional mPW1PW91 (which is based on the Perdew–Wang exchange, as modified by Adamo and Barone combined with PW91 correlation [30]). The latter was employed in combination with Pople’s triple-zeta basis set 6-311G(*d*) [31]. The calculated proton and carbon isotropic magnetic shielding constants were then converted into the ^1^H and ^13^C NMR chemical shifts scale, as recommended by IUPAC [32].

To take into account the systematic errors of the calculated chemical shifts, we have established correlations between their isotropic magnetic shielding constants (*y*) and experimental chemical shifts (*x*). Resulting linear regressions were further used to define the equations of the *y* = *a*x + *b* type. The slope *a* and intercept *b* were used then for recalculating unscaled shielding constants (*σ_calc_*) into their scaled values of chemical shifts (*δ_s_*) as *δ_s_* = (*σ_calc_* − *b*)/*a*.

### 3.3. Topological Analysis

The wave functions obtained as a result of geometry optimization were used for the further calculations to carry out the topological analysis of the real space functions. An analysis of the electron density using the obtained wave functions was performed with the AIMAll 19 program [33]; the latter used to locate and visualize the (3,‒1) critical points in the space of that descriptor.

### 3.4. Estimation of Probability Distribution of Diastereomers

To analyze the distribution of stereoisomers/conformers, we used the DP4+ approach, which was developed and introduced into practice by Sarotti [13,34]. The DP4+ formalism involves the inclusion of the unscaled chemical shifts in the probability calculation in combination with the implementation of the scaled chemical shifts:

(1) $$P_{i}=\frac{\prod_{k=1}^{N}\left[1-T_{s}^{\nu }\left(\frac{e_{s,k}^{i}}{\sigma_{s}}\right)\right]\left[1-T_{u-spx}^{\nu }\left(\frac{e_{u,k}^{i}-\mu_{u-spx}}{\sigma_{u-spx}}\right)\right]}{\sum_{j=1}^{m}\prod_{k=1}^{N}\left[1-T_{s}^{\nu }\left(\frac{e_{s,k}^{i}}{\sigma_{s}}\right)\right]\left[1-T_{u-spx}^{\nu }\left(\frac{e_{u,k}^{i}-\mu_{u-spx}}{\sigma_{u-spx}}\right)\right]}$$

where *T_s_^ν^* and *T_u−spx_^ν^* are the standard cumulative *t*-distribution function with *ν* degrees of freedom centered on the average error *μ* and variance *σ*, corresponding to the scaled and unscaled shielding constants, taking into account the hybridization of carbons. The scaled and unscaled errors *e_s_*, *e_u_* for nucleus *k* can be evaluated as *e_s,u_* = *δ_s,u_* − *δ_exp_*.

Thus, the configuration at each of the asymmetric center of alasmontamine A was established and verified by applying the DP4+ analysis of the probability of each of the candidate stereoisomers/conformers.

Mean Absolute Errors (MAE) were evaluated as:

(2) $$\mathrm{MAE}=\frac{\sum_{i=1}^{n}\left|e_{i}\right|}{n}$$

where *e_i_* is the error for each of *n* nuclei.

Corrected Mean Absolute Errors (CMAE) were calculated as:

(3) $$\mathrm{CMAE}=\frac{\sum_{i=1}^{n}\left|\delta_{exp}-\left(\frac{\sigma_{calc}-b}{a}\right)\right|}{n}$$

where *σ_calc_* are the unscaled shielding constants for each of the *n* nuclei, and *a* and *b* are the slope and intercept of the linear regression *σ_calc_* = *aδ_exp_* + *b*.

The Root-Mean-Square Deviations (RMSD) were evaluated as:

(4) $$\mathrm{RMSD}=\sqrt{\frac{\sum_{i=1}^{n}{\left(\delta_{exp}-\delta_{calc}\right)}^{2}}{n}}$$

while Pearson’s correlation coefficients *r* was calculated as:

(5) $$r(\delta_{exp},\delta_{calc})=\frac{\sum_{i=1}^{n}\left(\left(\delta_{exp}-\frac{\sum_{i=1}^{n}\delta_{exp}}{n}\right)\left(\delta_{calc}-\frac{\sum_{i=1}^{n}\delta_{calc}}{n}\right)\right)}{\sqrt{\sum_{i=1}^{n}{\left(\delta_{exp}-\frac{\sum_{i=1}^{n}\delta_{exp}}{n}\right)}^{2}\sum_{i=1}^{n}{\left(\delta_{calc}-\frac{\sum_{i=1}^{n}\delta_{calc}}{n}\right)}^{2}}}$$

where *δ_exp_* and *δ_calc_* are the experimental and scaled chemical shifts of each of the *n* nuclei, respectively.

The normalized values of the considered descriptors (*D*) were evaluated as N(*D*) = (*D*)/Range (*D*) × 100%.

## 4. Conclusions

A stereochemical structure of alasmontamine A was proposed in liquid phase based on the high-level DFT calculations of its ^1^H and ^13^C NMR chemical shifts. Six low energy conformers that contribute to the actual conformation of this alkaloid were identified, and three key configurations that define the resulting NMR shielding constants (and chemical shifts) were established. At that, several ambiguities in the reported assignment of the NMR chemical shifts of alasmontamine A were resolved. It was also shown that a previously proposed workflow for the stereochemical analysis of natural products [22] could be effectively applied to the calculation of ^1^H and ^13^C NMR chemical shifts of very large natural products consisting of more than 100 atoms of the second row elements, including those with multiple asymmetric centers and lone pairs exemplified here with alasmontamine A.

## Figures and Tables

**Table 1:** Selected ambiguous cases of calculated NMR chemical shifts (ppm) of the key configurations 0, 15′, and 15‴ of alasmontamine A. The errors of the performed calculations are given in parentheses.

Nucleus	0	15′	15‴	Exp.
^1^H NMR				
H-3*α*	3.97 (0.29)	4.00 (0.26)	3.98 (0.28)	4.26
H-9	7.15 (0.41)	7.24 (0.32)	7.15 (0.41)	7.56
H-18*α*	4.06 (−0.23)	4.12 (−0.29)	4.09 (−0.26)	3.83
H-21	4.18 (0.31)	4.17 (0.32)	4.15 (0.34)	4.49
H-17″*β*	2.36 (0.30)	2.41 (0.25)	2.35 (0.31)	2.66
H-23″*α*	4.36 (0.37)	4.29 (0.44)	4.36 (0.37)	4.73
^13^C NMR				
C-11′	114.2 (2.3)	114.0 (2.5)	114.1 (2.4)	116.5
C-21′	62.6 (2.1)	69.7 (−5.0)	62.6 (2.1)	64.7
C-22′	31.5 (−2.2)	32.1 (−2.8)	31.8 (−2.5)	29.3
C-15″	84.6 (2.5)	84.3 (2.8)	84.5 (2.6)	87.1
C-22″	166.5 (2.1)	166.5 (2.1)	166.5 (2.1)	168.6
C-7‴	58.1 (−2.1)	58.4 (−2.4)	57.8 (−1.8)	56.0
C-8‴	137.8 (−1.9)	138.1 (−2.2)	135.8 (0.1)	135.9
C-9‴	120.0 (−4.1)	119.7 (−3.8)	120.2 (−4.3)	115.9
C-11‴	124.1 (−5.2)	121.7 (−2.8)	124.2 (−5.3)	118.9
C-12‴	138.7 (3.3)	138.1 (3.9)	138.3 (3.7)	142.0
C-13‴	141.2 (−4.3)	140.7 (−3.8)	141.2 (−4.3)	136.9
C-21‴	62.3 (2.4)	62.3 (2.4)	69.5 (−4.8)	64.7
C-23‴	43.1 (2.1)	43.0 (2.2)	43.2 (2.0)	45.2

## Data Availability

Not applicable.

## Supplementary Materials

The following supporting information can be downloaded at: https://www.mdpi.com/article/10.3390/ijms24065572/s1.

## Abbreviations

BCP	Bond Critical Point
CMAE	Corrected Mean Absolute Error
COSY	COrrelated SpectroscopY
DFT	Density Functional Theory
HMBC	Heteronuclear Multiple Bond Correlation
HOHAHA	HOmonuclear HArtmann–HAhn spectroscopy
HSQC	Heteronuclear Single Quantum Coherence
IEF-PCM	Equation Formalism Polarizable Continuum Model
IUPAC	International Union of Pure and Applied Chemistry
MAE	Mean Absolute Error
NCMAE	Normalized Corrected Mean Absolute Error
NMR	Nuclear Magnetic Resonance
NOESY	Nuclear Overhauser Effect SpectroscopY
NRMSD	Normalized Root-Mean-Square Deviation
PES	Potential Energy Surface
PBE	Perdew–Burke–Ernzerhof (functional)
QTAIM	Atoms in Molecules
RMSD	Root-Mean-Square Deviation: A Quantum Theory
