# Supplementary material for: Stereochemical Study of the Super Large Tetrakis Alkaloid Alasmontamine A by Means of an Advanced Computational NMR
Source: Int J Mol Sci. 2023 Mar 14;24(6):5572. doi: 10.3390/ijms24065572 (PMC10054784; doi:10.3390/ijms24065572)

Supplementary Materials

Stereochemical Study of the Super Large Tetrakis Alkaloid Alasmontamine A by Means of an Advanced Computational NMR

TABLE OF CONTENTS

Cartesian coordinates (Angstroms) of key configurations of alasmontamine A optimized at the M06-2X/aug-cc-pVDZ level in the liquid phase of methanol, modulated within the IEF-PCM scheme S3

Compound: diastereomer «0» S3

Compound: diastereomer «15′» S9

Compound: diastereomer «15′′» S14

Figure S1. Integration probability distribution (%) of alternate diastereomers based on CMAE data.. S19

Cartesian coordinates (Angstroms) of key configurations of alasmontamine A optimized at the M06-2X/aug-cc-pVDZ level in the liquid phase of methanol, modulated within the IEF-PCM scheme:

Compound: diastereomer «0»

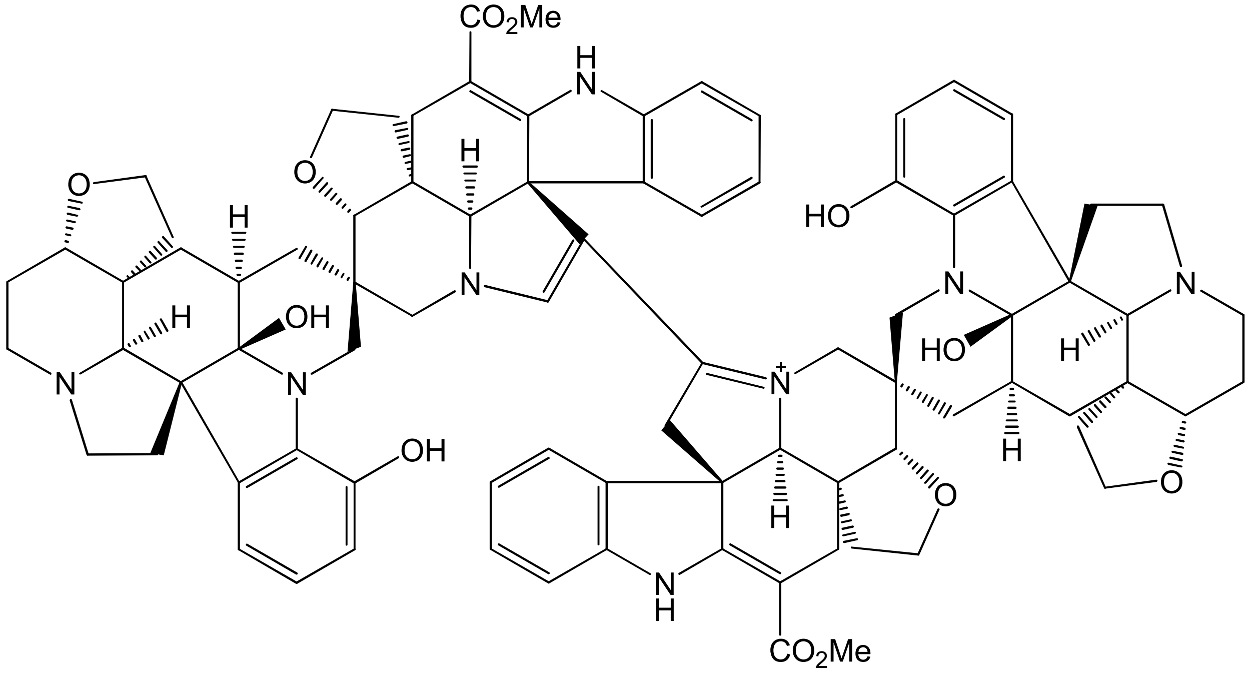

Imaginary frequencies: none

E^0: -4595.1873024 a.u.

N	-6.469236000	-0.974674000	2.483841000
C	-5.993793000	-0.541149000	1.279537000
C	-1.767896000	1.264250000	-0.604795000
N	-2.648759000	0.327001000	0.094932000
C	-2.574431000	-0.993994000	-0.000847000
C	-3.817697000	-1.614627000	0.569250000
C	-4.478135000	-0.462855000	1.374619000
C	-4.239007000	-0.636317000	2.856495000
C	-3.077322000	-0.572441000	3.610159000
C	-3.155101000	-0.844527000	4.982488000
C	-4.377154000	-1.183550000	5.566640000
C	-5.552922000	-1.260039000	4.811748000
C	-5.456906000	-0.979425000	3.454591000
C	-2.510989000	2.534253000	-1.072824000

C	-3.950993000	2.125250000	-1.450755000
C	-6.620978000	-0.183074000	0.126588000
C	-5.755026000	0.440004000	-0.938723000
C	-5.810148000	3.512329000	-1.038860000
C	-5.577631000	2.682279000	0.217835000
C	-4.787689000	1.479767000	-0.323530000
C	-3.884009000	0.816329000	0.735610000
C	-8.065118000	-0.238254000	-0.070252000
C	-10.148213000	-0.858520000	0.838669000
N	-0.585709000	3.708290000	-2.251081000
C	-0.606098000	4.825985000	-1.298859000
C	0.902731000	8.581462000	0.343870000
N	0.586288000	7.472008000	-0.546542000
C	1.159255000	7.633013000	-1.885924000
C	1.214904000	6.205985000	-2.456628000
C	0.871916000	5.277372000	-1.255018000
C	1.562594000	3.941604000	-1.427529000
C	2.885049000	3.584149000	-1.212455000
C	3.317246000	2.322201000	-1.632380000
C	2.432165000	1.459776000	-2.275748000
C	1.100062000	1.822550000	-2.507449000
C	0.654388000	3.073788000	-2.067658000
C	0.270747000	8.350272000	1.708270000
C	0.640490000	6.992195000	2.279427000
C	-1.062003000	4.288936000	0.072670000
C	-0.949626000	5.374390000	1.133153000
C	2.500999000	5.636432000	2.640024000
C	1.395179000	4.780600000	2.001959000
C	0.505205000	5.816204000	1.297996000
C	1.137148000	6.201877000	-0.051819000
C	-2.456684000	3.672337000	-0.033681000
C	-1.869186000	3.037665000	-2.390562000
N	-3.678023000	-4.307303000	-1.052576000
C	-2.422541000	-4.175071000	-0.526811000
C	2.004207000	-2.242752000	-1.384805000
N	0.557777000	-2.255524000	-1.256573000
C	-0.199619000	-1.330688000	-0.699243000
C	-1.526445000	-1.727650000	-0.572032000
C	-1.693215000	-3.074686000	-1.305375000
C	-2.637998000	-2.852000000	-2.473532000
C	-2.541846000	-2.003135000	-3.566257000
C	-3.635661000	-1.901079000	-4.434624000
C	-4.805243000	-2.623975000	-4.184275000
C	-4.920002000	-3.465618000	-3.073152000

C	-3.816263000	-3.566494000	-2.232893000
C	2.643174000	-3.420529000	-0.624732000
C	1.856796000	-4.704485000	-0.929868000
C	-1.787453000	-4.884753000	0.442804000
C	-0.297271000	-4.668833000	0.556771000
C	1.150958000	-6.090808000	-2.634559000
C	-0.017648000	-5.952633000	-1.643056000
C	0.313270000	-4.669664000	-0.856135000
C	-0.207108000	-3.450743000	-1.638565000
C	-2.413096000	-5.944022000	1.228849000
C	-4.400829000	-7.097082000	1.746049000
N	3.630242000	-2.254913000	1.409745000
C	5.002806000	-2.488867000	0.944275000
C	9.349836000	-2.103978000	1.194086000
N	7.930568000	-2.178373000	1.516332000
C	7.658204000	-1.982483000	2.943208000
C	6.191685000	-1.524367000	2.998491000
C	5.786112000	-1.283099000	1.514196000
C	4.717452000	-0.214260000	1.462546000
C	4.818781000	1.162265000	1.589997000
C	3.651571000	1.918855000	1.743186000
C	2.415380000	1.280122000	1.817493000
C	2.310300000	-0.109133000	1.717481000
C	3.466650000	-0.866509000	1.493606000
C	9.559219000	-2.322937000	-0.297551000
C	8.690857000	-1.400372000	-1.134597000
C	4.999326000	-2.479116000	-0.596298000
C	6.418564000	-2.537106000	-1.140570000
C	8.098885000	0.837564000	-1.351248000
C	6.812065000	-0.004620000	-1.390097000
C	7.219826000	-1.313921000	-0.693991000
C	7.158048000	-1.130119000	0.832069000
C	4.095722000	-3.597958000	-1.102694000
C	2.627814000	-3.186887000	0.906116000
H	-7.459165000	-1.053624000	2.675490000
H	-0.914149000	1.520225000	0.042139000
H	-1.380028000	0.726734000	-1.483079000
H	-3.588129000	-2.481442000	1.200993000
H	-4.464119000	-1.957383000	-0.252140000
H	-2.121504000	-0.312537000	3.148143000
H	-2.256319000	-0.793909000	5.596605000
H	-4.421034000	-1.393494000	6.635907000
H	-6.505914000	-1.523123000	5.270771000
H	-3.871031000	1.415720000	-2.294781000

H	-6.396218000	0.933730000	-1.680972000
H	-5.170155000	-0.311089000	-1.499228000
H	-5.849540000	4.591027000	-0.837044000
H	-6.737220000	3.216656000	-1.555100000
H	-4.981559000	3.243443000	0.949694000
H	-6.517675000	2.384220000	0.700389000
H	-3.616912000	1.535608000	1.520064000
H	-10.512564000	-1.314759000	1.763735000
H	-10.412108000	-1.479456000	-0.025848000
H	-10.573067000	0.144502000	0.711691000
H	2.003616000	8.683597000	0.443820000
H	0.519688000	9.511658000	-0.099943000
H	2.179820000	8.055563000	-1.813601000
H	0.545875000	8.322685000	-2.482638000
H	2.223428000	5.973238000	-2.818939000
H	0.507950000	6.047062000	-3.279198000
H	3.576905000	4.277712000	-0.730092000
H	4.347000000	2.008072000	-1.464048000
H	2.780381000	0.490292000	-2.640181000
H	0.580536000	9.136425000	2.411404000
H	-0.824712000	8.401968000	1.618128000
H	0.012643000	6.778631000	3.165221000
H	-0.345616000	3.495977000	0.348729000
H	-1.325969000	5.000534000	2.098286000
H	-1.581349000	6.233653000	0.854080000
H	3.430658000	5.624345000	2.050237000
H	2.733953000	5.307921000	3.663524000
H	1.800605000	4.033259000	1.306897000
H	0.812089000	4.257269000	2.774785000
H	2.237138000	6.304283000	0.083170000
H	-2.750022000	3.315951000	0.965077000
H	-3.172376000	4.454712000	-0.329749000
H	-2.558137000	3.778031000	-2.817285000
H	-1.771022000	2.212137000	-3.104195000
H	-4.326221000	-5.013225000	-0.729122000
H	2.256601000	-2.317803000	-2.454753000
H	2.365094000	-1.277489000	-1.003449000
H	0.258305000	-0.408786000	-0.343300000
H	-1.633108000	-1.423106000	-3.743969000
H	-3.577671000	-1.249591000	-5.306251000
H	-5.651429000	-2.529337000	-4.865692000
H	-5.836199000	-4.021253000	-2.873879000
H	2.214984000	-5.498538000	-0.245498000
H	0.141308000	-5.473514000	1.161396000

H	-0.086128000	-3.718387000	1.070294000
H	0.856627000	-5.905686000	-3.675986000
H	1.614914000	-7.086126000	-2.569828000
H	-0.988239000	-5.895018000	-2.153420000
H	-0.055603000	-6.804926000	-0.950045000
H	-0.086529000	-3.607751000	-2.720380000
H	-4.334117000	-6.874228000	2.817968000
H	-3.947672000	-8.075671000	1.547137000
H	-5.443221000	-7.081077000	1.415160000
H	9.754119000	-1.115575000	1.497730000
H	9.883907000	-2.874050000	1.769300000
H	8.319525000	-1.194737000	3.352541000
H	7.851644000	-2.911353000	3.498375000
H	6.103402000	-0.582328000	3.552789000
H	5.527660000	-2.261485000	3.464781000
H	5.797273000	1.647622000	1.579782000
H	3.711060000	3.004254000	1.825416000
H	1.494595000	1.842595000	1.982050000
H	10.614762000	-2.165574000	-0.561354000
H	9.307763000	-3.363077000	-0.554429000
H	8.717529000	-1.725559000	-2.192151000
H	4.573136000	-1.507086000	-0.902319000
H	6.397886000	-2.576973000	-2.240871000
H	6.909982000	-3.462157000	-0.796129000
H	8.292818000	1.327579000	-2.316676000
H	8.070062000	1.609447000	-0.567807000
H	6.514744000	-0.216994000	-2.427706000
H	5.976824000	0.502186000	-0.889076000
H	7.575586000	-0.131692000	1.091317000
H	4.117069000	-3.633565000	-2.200747000
H	4.477252000	-4.564587000	-0.737872000
H	1.648108000	-2.829358000	1.229826000
H	2.811839000	-4.153788000	1.397870000
H	-1.215286000	6.645663000	-1.339545000
H	0.720655000	0.274047000	-3.580442000
H	6.418206000	-3.706002000	1.394286000
H	1.023901000	-1.209556000	2.655127000
O	-3.737376000	-6.077741000	0.987656000
O	5.445555000	-3.733053000	1.439041000
O	9.172803000	-0.060152000	-1.039655000
O	1.054558000	-0.690449000	1.839878000
O	-8.650081000	0.156418000	-1.064625000
O	-4.692911000	3.254161000	-1.898791000
O	-1.491034000	5.816241000	-1.769934000

O	2.016509000	6.986108000	2.663244000
O	-8.724308000	-0.781886000	0.978978000
O	0.224959000	0.995703000	-3.173329000
O	2.109813000	-5.088918000	-2.276277000
O	-1.830740000	-6.649323000	2.034400000

Compound: diastereomer «15′»

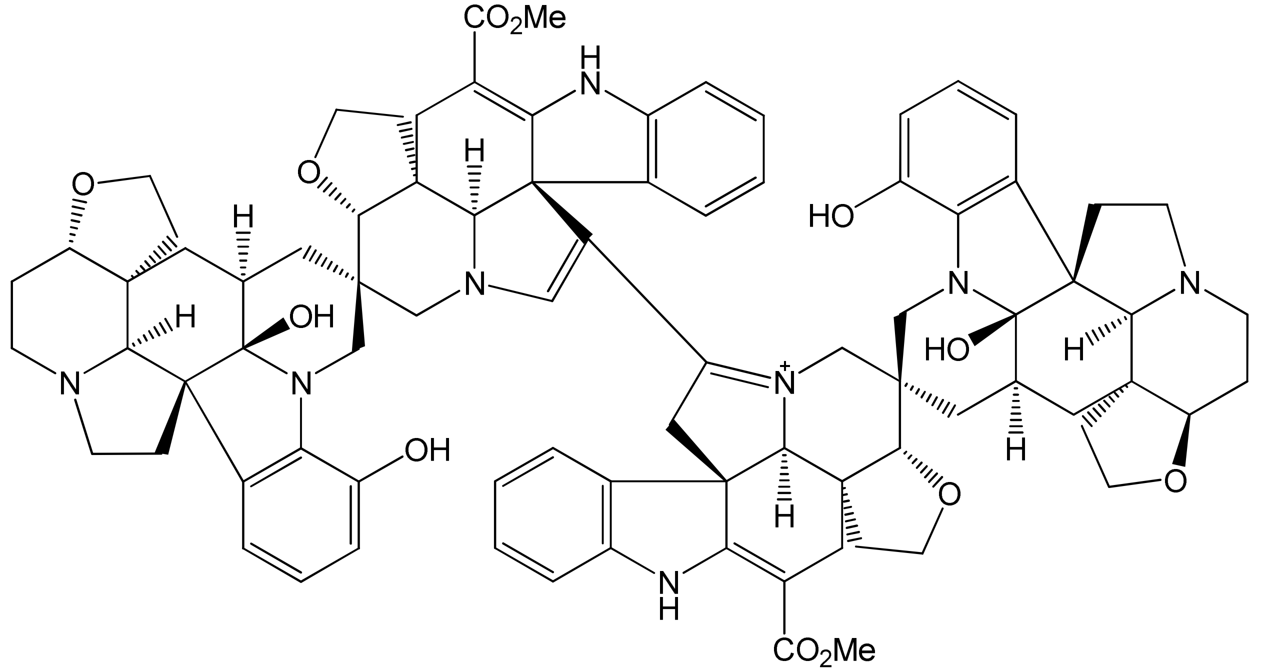

Imaginary frequencies: none

E^0: -4595.1785102 a.u.

N	-6.324883000	-1.117801000	2.492651000
C	-5.873088000	-0.665000000	1.286331000
C	-1.653724000	1.192790000	-0.575915000
N	-2.535875000	0.230403000	0.085443000
C	-2.461987000	-1.088791000	-0.047047000
C	-3.709996000	-1.722436000	0.500300000
C	-4.356196000	-0.594613000	1.349649000
C	-4.083722000	-0.806701000	2.820774000
C	-2.903870000	-0.770771000	3.547145000
C	-2.950696000	-1.078055000	4.913740000
C	-4.161353000	-1.422851000	5.518256000
C	-5.355761000	-1.470135000	4.790499000
C	-5.290516000	-1.154934000	3.438934000
C	-2.414596000	2.442271000	-1.076156000
C	-3.850146000	2.007352000	-1.451208000
C	-6.521461000	-0.279263000	0.154193000
C	-5.670861000	0.347550000	-0.921879000
C	-5.689473000	3.419818000	-1.025171000
C	-5.458533000	2.583861000	0.230021000
C	-4.682960000	1.373859000	-0.315238000
C	-3.774282000	0.700714000	0.733136000
C	-7.970378000	-0.308312000	-0.008464000
C	-10.040475000	-0.912043000	0.939094000
N	-0.526372000	3.658682000	-2.276528000

C	-0.620896000	4.809878000	-1.372020000
C	0.337391000	8.679953000	0.180226000
N	0.287188000	7.499828000	-0.676419000
C	0.814788000	7.722359000	-2.025717000
C	1.084396000	6.303891000	-2.565657000
C	0.825014000	5.363834000	-1.356017000
C	1.606614000	4.078009000	-1.493297000
C	2.956196000	3.831339000	-1.295405000
C	3.473261000	2.586223000	-1.667659000
C	2.640249000	1.624825000	-2.236617000
C	1.280489000	1.878126000	-2.455514000
C	0.753488000	3.117367000	-2.076412000
C	-0.204880000	8.373399000	1.581494000
C	0.579195000	7.183761000	2.080390000
C	-1.032627000	4.294589000	0.021751000
C	-0.981260000	5.416927000	1.054699000
C	0.882175000	5.426625000	3.498980000
C	1.282438000	4.984700000	2.068687000
C	0.461124000	5.938848000	1.191380000
C	1.026613000	6.321390000	-0.173594000
C	-2.392873000	3.600522000	-0.055869000
C	-1.772312000	2.919732000	-2.405190000
N	-3.547878000	-4.388238000	-1.182543000
C	-2.307323000	-4.259409000	-0.620929000
C	2.132988000	-2.311198000	-1.370662000
N	0.685525000	-2.327910000	-1.278598000
C	-0.082074000	-1.407858000	-0.729038000
C	-1.409577000	-1.809802000	-0.623002000
C	-1.558471000	-3.152151000	-1.369575000
C	-2.470209000	-2.920144000	-2.561611000
C	-2.341086000	-2.066217000	-3.646692000
C	-3.406244000	-1.964656000	-4.550238000
C	-4.580569000	-2.692980000	-4.341082000
C	-4.728560000	-3.539871000	-3.237715000
C	-3.652930000	-3.639679000	-2.361568000
C	2.757157000	-3.473587000	-0.574157000
C	1.992337000	-4.766394000	-0.898474000
C	-1.695733000	-4.976077000	0.358483000
C	-0.211581000	-4.750274000	0.520346000
C	1.331051000	-6.126462000	-2.652731000
C	0.154515000	-6.028721000	-1.668227000
C	0.445265000	-4.742046000	-0.872576000
C	-0.065556000	-3.527690000	-1.670420000
C	-2.336976000	-6.048992000	1.112788000

C	-4.332749000	-7.217462000	1.558912000
N	3.625446000	-2.242110000	1.475055000
C	5.022401000	-2.389993000	1.041176000
C	9.329242000	-1.621939000	1.353613000
N	7.915121000	-1.807101000	1.655372000
C	7.596482000	-1.559606000	3.063905000
C	6.091743000	-1.244076000	3.070677000
C	5.700254000	-1.102163000	1.569004000
C	4.554991000	-0.121182000	1.449318000
C	4.545234000	1.264096000	1.492737000
C	3.319076000	1.932709000	1.589515000
C	2.139066000	1.202956000	1.711550000
C	2.146673000	-0.194838000	1.703219000
C	3.358907000	-0.866415000	1.505197000
C	9.589583000	-1.899906000	-0.119907000
C	8.663007000	-1.101840000	-1.019934000
C	5.060959000	-2.444793000	-0.498818000
C	6.495615000	-2.431248000	-1.003629000
C	7.880156000	1.061241000	-1.364286000
C	6.675729000	0.104899000	-1.384364000
C	7.181843000	-1.123323000	-0.609379000
C	7.069535000	-0.867551000	0.903815000
C	4.230428000	-3.625027000	-0.991290000
C	2.694909000	-3.238297000	0.954018000
H	-7.310759000	-1.199305000	2.704025000
H	-0.836146000	1.475064000	0.107155000
H	-1.213870000	0.669450000	-1.436322000
H	-3.486244000	-2.612261000	1.100749000
H	-4.360333000	-2.030406000	-0.331694000
H	-1.957948000	-0.505036000	3.068961000
H	-2.037291000	-1.047802000	5.507080000
H	-4.181758000	-1.660110000	6.582406000
H	-6.299281000	-1.737922000	5.265548000
H	-3.760558000	1.286390000	-2.283858000
H	-6.321992000	0.853072000	-1.647054000
H	-5.102685000	-0.403502000	-1.498143000
H	-5.682154000	4.499652000	-0.824062000
H	-6.640186000	3.160659000	-1.516213000
H	-4.858811000	3.135886000	0.964645000
H	-6.400216000	2.291234000	0.712466000
H	-3.509678000	1.409098000	1.528543000
H	-10.390038000	-1.383809000	1.861814000
H	-10.337870000	-1.506574000	0.066964000
H	-10.447736000	0.101940000	0.846704000

H	1.382059000	9.048680000	0.265772000
H	-0.253451000	9.474889000	-0.296073000
H	1.755839000	8.303094000	-1.980268000
H	0.091945000	8.291171000	-2.627801000
H	2.130173000	6.201919000	-2.880703000
H	0.440087000	6.037194000	-3.411425000
H	3.603247000	4.597969000	-0.865183000
H	4.529427000	2.363579000	-1.518288000
H	3.049659000	0.660677000	-2.549312000
H	-0.050357000	9.244726000	2.232545000
H	-1.282301000	8.159269000	1.545714000
H	1.650049000	7.477573000	2.077583000
H	-0.272700000	3.546320000	0.303257000
H	-1.337067000	5.047297000	2.028224000
H	-1.669307000	6.219346000	0.749833000
H	0.159910000	4.741748000	3.963695000
H	1.761404000	5.510146000	4.154109000
H	2.356584000	5.150588000	1.898677000
H	1.067244000	3.923163000	1.895043000
H	2.108955000	6.557575000	-0.066391000
H	-2.651832000	3.240984000	0.951594000
H	-3.150582000	4.342752000	-0.348245000
H	-2.486652000	3.607034000	-2.876323000
H	-1.622782000	2.068952000	-3.079270000
H	-4.200288000	-5.102746000	-0.887173000
H	2.413815000	-2.406575000	-2.431469000
H	2.475390000	-1.335360000	-1.001680000
H	0.368606000	-0.483543000	-0.371245000
H	-1.428963000	-1.482634000	-3.792251000
H	-3.321184000	-1.309551000	-5.416752000
H	-5.404560000	-2.598569000	-5.048969000
H	-5.648091000	-4.100638000	-3.072003000
H	2.332725000	-5.555161000	-0.199499000
H	0.213874000	-5.552281000	1.137373000
H	-0.025522000	-3.798719000	1.041046000
H	1.044934000	-5.884471000	-3.685128000
H	1.787160000	-7.126859000	-2.638925000
H	-0.814078000	-6.000322000	-2.184404000
H	0.140762000	-6.883937000	-0.977692000
H	0.077366000	-3.688152000	-2.749008000
H	-4.269731000	-7.035684000	2.638435000
H	-3.887533000	-8.191852000	1.324987000
H	-5.372889000	-7.179546000	1.223182000
H	9.636961000	-0.586172000	1.608786000

H	9.917114000	-2.308193000	1.979909000
H	8.169690000	-0.688966000	3.436506000
H	7.866779000	-2.432053000	3.675162000
H	5.899096000	-0.295652000	3.585942000
H	5.492668000	-2.025121000	3.552853000
H	5.481045000	1.825714000	1.456934000
H	3.286853000	3.020958000	1.590991000
H	1.176463000	1.697537000	1.856655000
H	10.633232000	-1.667086000	-0.374269000
H	9.431975000	-2.969607000	-0.324165000
H	8.740669000	-1.478299000	-2.057681000
H	4.592689000	-1.510906000	-0.854426000
H	6.508059000	-2.532402000	-2.100022000
H	7.047181000	-3.294030000	-0.595409000
H	8.051607000	1.520134000	-2.348852000
H	7.765481000	1.864178000	-0.621051000
H	6.427915000	-0.189020000	-2.415043000
H	5.782441000	0.556160000	-0.932152000
H	7.393481000	0.175402000	1.118030000
H	4.297280000	-3.703957000	-2.085048000
H	4.631438000	-4.560066000	-0.568842000
H	1.687795000	-2.947756000	1.258135000
H	2.931959000	-4.191561000	1.449554000
H	-1.373699000	6.562036000	-1.446206000
H	0.990082000	0.259768000	-3.452458000
H	6.514724000	-3.475654000	1.577234000
H	1.010447000	-1.407714000	2.688682000
O	-3.656096000	-6.177064000	0.841925000
O	5.546423000	-3.574242000	1.599259000
O	9.023168000	0.279317000	-0.989903000
O	0.944719000	-0.861646000	1.893362000
O	-8.573516000	0.119683000	-0.977906000
O	-4.610248000	3.114968000	-1.919381000
O	-1.574860000	5.711956000	-1.880362000
O	0.247446000	6.714106000	3.377189000
O	-8.612058000	-0.866327000	1.044007000
O	0.452444000	0.956312000	-3.054981000
O	2.294218000	-5.150867000	-2.235083000
O	-1.771539000	-6.769887000	1.916659000

Compound: diastereomer «15‴»

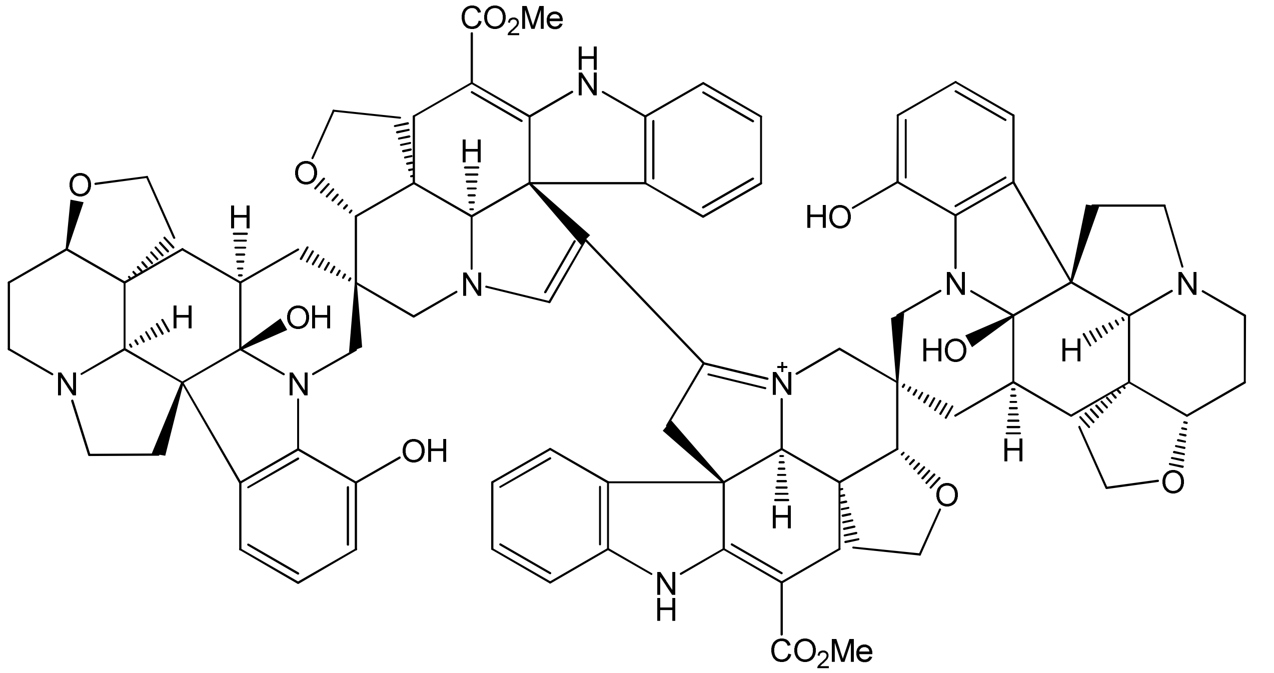

Imaginary frequencies: none

E^0: -4595.1801423 a.u.

N	-6.443495000	-1.159540000	2.418538000
C	-5.982738000	-0.695756000	1.219573000
C	-1.780904000	1.229930000	-0.606550000
N	-2.650953000	0.257099000	0.055397000
C	-2.543084000	-1.061376000	-0.058590000
C	-3.787490000	-1.716088000	0.472661000
C	-4.468412000	-0.596407000	1.305665000
C	-4.214994000	-0.800046000	2.781437000
C	-3.047651000	-0.738974000	3.526028000
C	-3.109019000	-1.046446000	4.891943000
C	-4.321386000	-1.416481000	5.477766000
C	-5.503033000	-1.489695000	4.731499000
C	-5.423545000	-1.173787000	3.380906000
C	-2.553225000	2.479678000	-1.086837000
C	-3.980726000	2.036087000	-1.477974000
C	-6.622388000	-0.319064000	0.079565000
C	-5.769465000	0.329401000	-0.981751000
C	-5.853362000	3.404306000	-1.054504000
C	-5.616593000	2.560982000	0.193780000
C	-4.811621000	1.372684000	-0.356770000
C	-3.900710000	0.707949000	0.694296000
C	-8.068222000	-0.374292000	-0.103383000
C	-10.139849000	-1.020137000	0.812350000
N	-0.645254000	3.705368000	-2.240676000

C	-0.716370000	4.828644000	-1.297129000
C	0.642185000	8.648531000	0.324483000
N	0.381995000	7.517660000	-0.557463000
C	0.977835000	7.679407000	-1.886729000
C	1.084802000	6.247721000	-2.438230000
C	0.746422000	5.324446000	-1.231344000
C	1.478707000	4.007444000	-1.374466000
C	2.805036000	3.688097000	-1.125880000
C	3.281708000	2.434392000	-1.522811000
C	2.435676000	1.543501000	-2.179916000
C	1.099973000	1.868620000	-2.446823000
C	0.608824000	3.108695000	-2.025577000
C	-0.009376000	8.411967000	1.679333000
C	0.394157000	7.074611000	2.274573000
C	-1.175844000	4.287864000	0.071962000
C	-1.121098000	5.389342000	1.120961000
C	2.284407000	5.782602000	2.698535000
C	1.223109000	4.884333000	2.042119000
C	0.314915000	5.882425000	1.307044000
C	0.961295000	6.270630000	-0.035057000
C	-2.545206000	3.620439000	-0.048468000
C	-1.906776000	2.996967000	-2.397489000
N	-3.508182000	-4.419181000	-1.097517000
C	-2.274567000	-4.235541000	-0.536055000
C	2.110186000	-2.175920000	-1.295224000
N	0.661984000	-2.235388000	-1.207085000
C	-0.142152000	-1.326017000	-0.692909000
C	-1.461216000	-1.762585000	-0.605432000
C	-1.557040000	-3.128926000	-1.316801000
C	-2.464654000	-2.971371000	-2.523945000
C	-2.349677000	-2.163685000	-3.645384000
C	-3.407417000	-2.135909000	-4.562861000
C	-4.560538000	-2.891365000	-4.333467000
C	-4.693924000	-3.693968000	-3.195660000
C	-3.625673000	-3.720456000	-2.305534000
C	2.767192000	-3.322075000	-0.501525000
C	2.029819000	-4.635913000	-0.804611000
C	-1.647655000	-4.902223000	0.468623000
C	-0.168243000	-4.640489000	0.621728000
C	1.415032000	-6.074170000	-2.501040000
C	0.214745000	-5.954807000	-1.545020000
C	0.484141000	-4.648382000	-0.772760000
C	-0.050101000	-3.461077000	-1.592983000
C	-2.265804000	-5.961724000	1.260314000

C	-4.236358000	-7.155050000	1.750370000
N	3.661136000	-2.090712000	1.535778000
C	5.051931000	-2.284479000	1.107279000
C	9.332437000	-1.936851000	1.259431000
N	7.919251000	-1.905716000	1.622056000
C	7.681213000	-1.745935000	3.059287000
C	6.219420000	-1.266406000	3.151870000
C	5.780661000	-1.041333000	1.677388000
C	4.676470000	-0.015279000	1.608367000
C	4.731332000	1.363854000	1.729096000
C	3.536338000	2.083219000	1.843307000
C	2.319919000	1.405185000	1.881616000
C	2.261076000	0.012262000	1.788349000
C	3.447354000	-0.709349000	1.607312000
C	9.517472000	-2.079193000	-0.256279000
C	8.714521000	-0.965263000	-0.884379000
C	5.085873000	-2.298393000	-0.433462000
C	6.516343000	-2.294101000	-0.962077000
C	7.678918000	0.005674000	-2.665113000
C	6.768591000	0.169240000	-1.420533000
C	7.222490000	-0.996665000	-0.529009000
C	7.132731000	-0.828157000	0.985029000
C	4.237208000	-3.459092000	-0.941271000
C	2.704613000	-3.065648000	1.024565000
H	-7.430770000	-1.258073000	2.615581000
H	-0.957561000	1.509724000	0.070146000
H	-1.347878000	0.714681000	-1.476152000
H	-3.558762000	-2.598196000	1.082170000
H	-4.417838000	-2.039259000	-0.369217000
H	-2.100417000	-0.453511000	3.061977000
H	-2.205802000	-0.996724000	5.499365000
H	-4.353369000	-1.653547000	6.541672000
H	-6.447836000	-1.777346000	5.192179000
H	-3.878495000	1.330523000	-2.322224000
H	-6.420196000	0.823140000	-1.715333000
H	-5.176398000	-0.407800000	-1.550695000
H	-5.883317000	4.481258000	-0.841399000
H	-6.787096000	3.120701000	-1.564587000
H	-5.033179000	3.118474000	0.937635000
H	-6.555853000	2.245326000	0.666318000
H	-3.651754000	1.416929000	1.494109000
H	-10.493452000	-1.503734000	1.727364000
H	-10.414379000	-1.614574000	-0.067296000
H	-10.564088000	-0.013104000	0.720248000

H	1.736611000	8.789945000	0.444808000
H	0.236805000	9.558994000	-0.139660000
H	1.984684000	8.131000000	-1.799252000
H	0.358098000	8.344819000	-2.503930000
H	2.105922000	6.039917000	-2.779372000
H	0.398373000	6.057877000	-3.271469000
H	3.465608000	4.403816000	-0.632344000
H	4.313679000	2.148364000	-1.320495000
H	2.816435000	0.580518000	-2.528630000
H	0.258451000	9.216687000	2.378407000
H	-1.103979000	8.424146000	1.566746000
H	-0.242312000	6.852473000	3.152119000
H	-0.436397000	3.523869000	0.367228000
H	-1.502502000	5.015141000	2.084042000
H	-1.776995000	6.222509000	0.818886000
H	3.232807000	5.787383000	2.139447000
H	2.492948000	5.477819000	3.734427000
H	1.669053000	4.144800000	1.363994000
H	0.639341000	4.348473000	2.805499000
H	2.054798000	6.408006000	0.119504000
H	-2.834527000	3.253048000	0.947528000
H	-3.285185000	4.377163000	-0.350509000
H	-2.609402000	3.715358000	-2.839303000
H	-1.773859000	2.171495000	-3.105576000
H	-4.139460000	-5.144028000	-0.782122000
H	2.394470000	-2.254845000	-2.356741000
H	2.425405000	-1.193679000	-0.917098000
H	0.274807000	-0.384155000	-0.339803000
H	-1.453569000	-1.560843000	-3.809634000
H	-3.332850000	-1.517842000	-5.457096000
H	-5.378764000	-2.854770000	-5.053290000
H	-5.595923000	-4.278421000	-3.015449000
H	2.393426000	-5.406446000	-0.096815000
H	0.274506000	-5.421476000	1.253447000
H	0.001880000	-3.675660000	1.123280000
H	1.144173000	-5.918740000	-3.553347000
H	1.908605000	-7.052296000	-2.403810000
H	-0.742080000	-5.936637000	-2.083533000
H	0.183288000	-6.795534000	-0.837810000
H	0.113160000	-3.632133000	-2.666951000
H	-4.178368000	-6.930887000	2.822190000
H	-3.769947000	-8.127815000	1.553925000
H	-5.276748000	-7.152497000	1.413311000
H	9.836665000	-1.007694000	1.602162000

H	9.803799000	-2.779038000	1.785281000
H	8.363594000	-0.983332000	3.480981000
H	7.865728000	-2.694556000	3.583492000
H	6.159648000	-0.312635000	3.690348000
H	5.561177000	-1.987290000	3.650352000
H	5.693839000	1.878455000	1.746668000
H	3.559099000	3.169918000	1.921124000
H	1.377050000	1.939551000	2.010767000
H	10.583122000	-1.981837000	-0.505200000
H	9.174147000	-3.064435000	-0.601748000
H	9.117915000	-0.008960000	-0.489359000
H	4.614896000	-1.354271000	-0.754686000
H	6.506523000	-2.365275000	-2.060084000
H	7.046779000	-3.182710000	-0.589684000
H	7.145644000	-0.427166000	-3.522223000
H	8.116764000	0.967249000	-2.970435000
H	5.705796000	0.134231000	-1.692294000
H	6.961393000	1.129647000	-0.920096000
H	7.537519000	0.169823000	1.266574000
H	4.285521000	-3.512190000	-2.037635000
H	4.641482000	-4.406004000	-0.550713000
H	1.704295000	-2.739412000	1.316066000
H	2.911036000	-4.017267000	1.536631000
H	-1.375794000	6.629163000	-1.365908000
H	0.785089000	0.303898000	-3.519189000
H	6.488173000	-3.462983000	1.559814000
H	0.975357000	-1.110942000	2.699913000
O	-3.581728000	-6.128214000	0.994480000
O	5.517754000	-3.505947000	1.634332000
O	8.730817000	-0.908103000	-2.301119000
O	1.020176000	-0.607058000	1.875779000
O	-8.665629000	0.047502000	-1.079042000
O	-4.748548000	3.145061000	-1.931012000
O	-1.622351000	5.787714000	-1.790985000
O	1.762345000	7.118039000	2.682070000
O	-8.714039000	-0.949247000	0.937313000
O	0.263117000	1.015808000	-3.128119000
O	2.330894000	-5.034907000	-2.136649000
O	-1.685497000	-6.640594000	2.089764000

Figure S1. Integration probability distribution (%) of alternate diastereomers based on CMAE data.

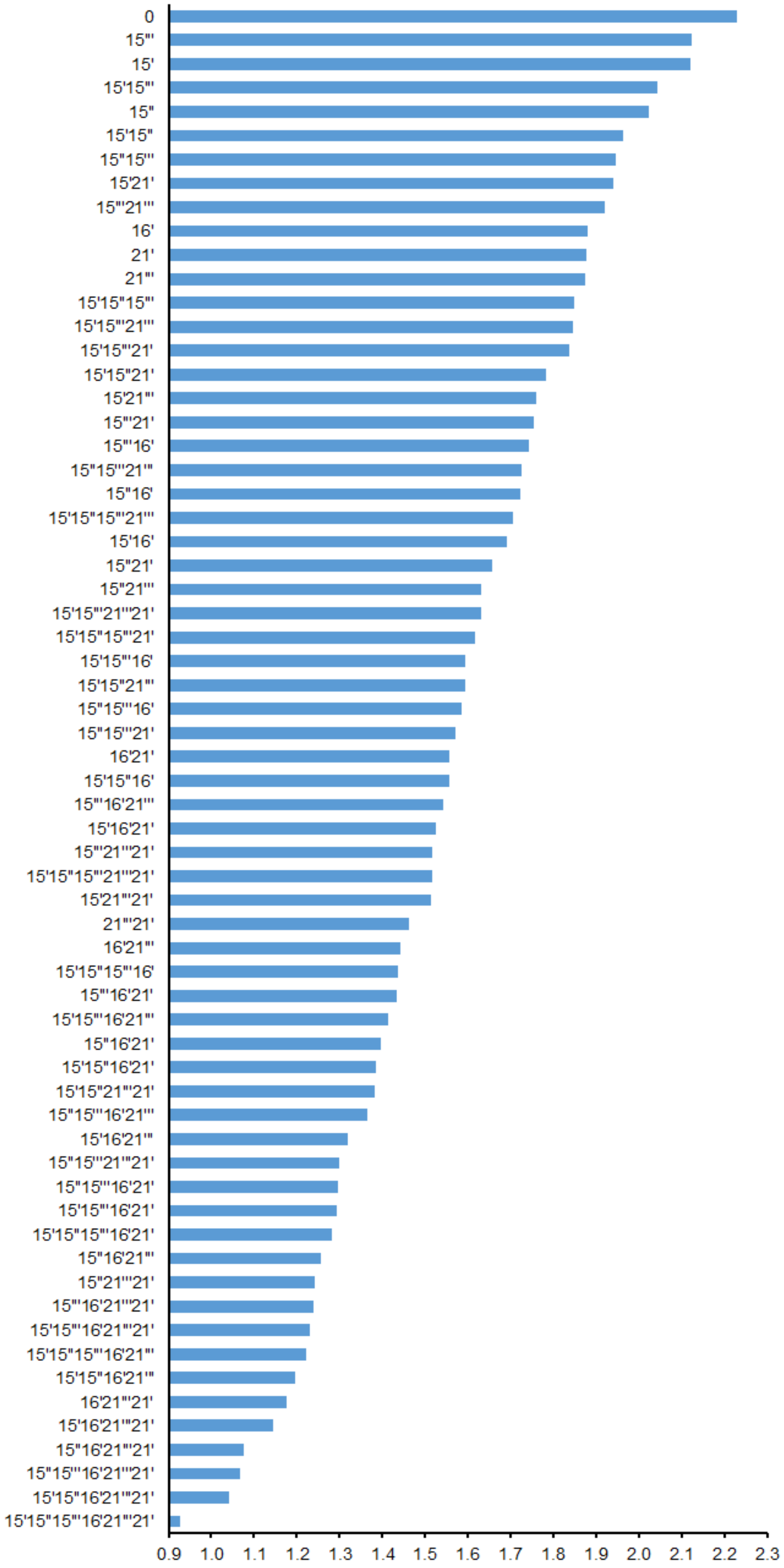

Supplement: Supplementary file 1 [file ijms-24-05572-s001.zip › Supplementary Materials S1.pdf]
